# Hospital News

**Published:** 1922-10

**Authors:** 


### HOSPITAL NEWS.

In August, 1,269 patients were waiting admission to the Edinburgh Royal Infirmary, the highest figure yet recorded.

The Towyn War Memorial Cottage Hospital has been opened. It has eight beds and a theatre, and cost £8,000.

Dr. R. W. Wilson, on his retirement from the post of Medical Superintendent of Croydon Infirmary after 37 years' service, has been presented with a clock, and also a china tea service. The latter was a gift from the patients.

Mr. G. J. Allen, chairman of the board of management of Croydon Hospital, has announced a scheme of extension which will add 100 beds, including paying wards, and a new out-patient department.

A new nurses' home is being built at the Nottingham General Hospital on high ground close by, overlooking the Castle precincts. The home contains eight sitting-rooms and about 130 other rooms.

The foundation stone has been laid of the Brompton and District War Memorial Cottage Hospital, Cumberland. A gift of £500, in addition to the £5,000 in hand, has named the " Lacey Thompson " bed after the donor.

The new Maternity Home and Children's Hospital, built by the Corporation in conjunction with the Ministry of Health, has been opened at Chesterfield. There are 18 beds, an ante-natal clinic, a child welfare centre, and twelve cots in the infants' hospital.

The King Edward VII. Welsh National Memorial Association has converted Craig-y-Nos Castle, on which its former owner, Madame Patti, is said to have spent £100,000, into a sanatorium for 104 consumptives. The castle was bought for £19,000, but a large sum had to be spent upon alterations and repairs.

The Committee of the Chelsea Hospital has decided to invite not more than 12 architects to submit designs for the projected nurses' home, with 100 rooms or so. Mr. Henry V. Ashley, F.R.I.B.A., has been chosen to judge the competition ; and premiums of £150, £100 and £50 will be given to the architects whose plans are placed first, second and third upon the list.

On October 4 the War Memorial Cottage Hospital at Burnham-on-Sea, Somerset, is to be opened by the Marquess of Bath. The cost of the building, £3,300, has been met except for £65, and a concession has been made to ex-service patients in return for their gift of £100, subscribed for their benefit. A nurse has been appointed to the Committee of Management in the person of Nurse Elia Scott. The names of the fallen are to be placed inside the hospital. Mr. Fay is the architect.

A most interesting exhibit at the Manchester Health and Housing Exhibition was that of the Salford Royal Hospital. Its first stand depicted the child welfare department, and how a baby in a poor home can be tended if the parents have a proper pride. The hospital sisters combined instruction with entertainment in their explanations. A second stand displayed the electrical appliances used in the hospital's clinics. Much interest was shown in both, and the hospital has made the most of the publicity afforded by the exhibition.

The Senior Tuberculosis Officer for Manchester, Dr. D. P. Sutherland, M.B., B.Sc., strongly supports the Barrowmere Tuberculosis Colony, where graduated labour and self-supporting work under medical supervision are secured for infective cases. Funds are asked to enable the village settlement to be realised. In the spring of 1921 the first patients were admitted to Barrowmere Hall, and 60 men are now working there. A start was made in an army hut, which grew into a carpenter's shop, from which the estate has been provided with essentials for other men's trades—coops, lockers, pig-houses, doors, window-panes, open-air shelters. The poultry farm, employing 12 men, has 600 laying birds. It is because the grant of £37,000 from the Ministry of Health has been reduced to £7,500 that the appeal is made.

The Ministry of Health has prevented the Chiswick and Ealing Hospital Committee from refusing admission to the maternity hospital to unmarried mothers.

By Royal permission, the Portsmouth Naval Maternity Home is now to be known as the Royal Naval Maternity Home, Portsmouth.

The Whickham and District War Memorial Cottage Hospital, which has now been formally opened, contains at present 12 beds (there is room for 24), maternity and dental clinics. Mr. F. W. Morrison is chairman of the committee.

A memorial tablet to Dr. Edward Mansel Sympson, for 30 years surgeon to the hospital, has been unveiled at Lincoln County Hospital. Dr Sympson was also editor of " Lincolnshire Notes and Queries."

Because in previous years there had not been sufficient rain to justify a claim, the Committee of the Leeds General Hospital Gala, which was spoilt through a torrential downpour, had decided this year not to insure against wet weather.

The foundation stone of the new out-patient department at the Royal Gwent Hospital has been laid by Sir Garrod Thomas. Messrs. Griggs & Vaughan are the architects of the new wing, which is costing £46,000. The department is part of an important extension scheme.

The new Maternity Home of the Exeter and District Nursing Association has been opened in the city. The new accommodation provides for 15 patients instead of six. The former home, started in 1918, still houses the staff. The institution is a recognised training school for midwives, and contains wards for two private patients.

To the two closed wards at King's College Hospital that were reopened for paying patients, two more have been added. Another empty ward is being provided with a deep therapy apparatus under Dr. Robert Knox, the senior radiologist, for the benefit chiefly of private patients likely to gain relief from its use.

It is announced that all except 100 beds are being closed at the Royal Sussex County Hospital, Brighton. The full complement of beds is 220. It will be recalled that the so-called Sussex scheme for giving privileges to subscribers was designed to save the situation. A sum of £15,000 is needed before the end of the year, hence the recent decision partly to close the hospital.

The governors of the Sunderland Infirmary have decided to spend £2,000 on additional accommodation for the office, dispensary, and nursing staff. It has been found essential to have the offices " as near as possible to the main building." Hitherto they have been too far away, and the best rule is, of course, to make the administration's office the main building itself. Compared with this change, the spending of £1,000 also upon a new X-ray installation must be regarded as a secondary improvement, for developments must begin from an appropriate centre, the hub of which is the administration block.

The Commissioners of the Board of Control have criticised the milking arrangements at the farm attached to Napsbury Mental Hospital. The Commissioners report : " The number of overalls appeared to us insufficient and not clean enough in use . . . a reserve of clean garments should always be available for use whenever soiling renders an additional change necessary. We had no means of discovering whether the cows are in any way cleansed and prepared for milking, but the condition of the overalls shown to us seemed to indicate that little is done in this direction. We were also extremely dissatisfied with the state as to cleanliness of the milking pails, which were obviously placed ready for the next milking. They had been insufficiently cleansed, and the interior sides of nearly all of them were covered with a flaky deposit, the product of previous milkings. We think that the attention of the farm bailiff should be drawn to the Board's circular of May, 1920, containing suggestions for the prevention of the spread of disease by milk."

